# Supplementary material for: Trophic Niche Breadth of Falconidae Species Predicts Biomic Specialisation but Not Range Size
Source: Biology (Basel). 2022 Mar 29;11(4):522. doi: 10.3390/biology11040522 (PMC9028249; doi:10.3390/biology11040522)
Supplement: Supplementary file 1 [file biology-11-00522-s001.zip › biology-1610510-supplementary.pdf]

SUPPLEMENTARY MATERIALS

Trophic niche breadth of Falconidae species predicts biomic specialisation but not range size

Juan A. Fargallo, Juan Navarro-López, Juan L. Cantalapiedra, Jonathan S. Pelegrin,

Manuel Hernández Fernández

Table S1. Variance inflation factor (VIF) values of diet variables used for range size and BSI models.

Diet variable	**VIF**
Diet richness (class)	2.95
Diet richness (order)	4.35
Diet diversity (class)	11.49
Diet diversity (order)	7.52
Maximum diet diversity (class)	12.20
Maximum diet diversity (order)	12.19

Table S2. Classification of biomes inhabited by Falconidae. Names and typology of biomes (Walter 1970), number and percentage of Falconidae species occupying each biome and number of specialist species (BSI = 1) in each biome are shown.

Biome	Typolo gy	Number of species	Percentage of species	Number of specialists
Evergreen tropical rainforest	I	26	42.6	5
Tropical deciduous woodland	II	38	62.3	2
Savannah	II/III	30	49.2	2
Sub-Tropical desert	III	21	34.4	2
Sclerophyllous woodland and shrubland	IV	18	29.5	1
Temperate evergreen forest	V	22	36.1	0
Broad-leaf deciduous forest	VI	17	27.9	0
Steppe/cold desert	VII	18	29.5	2
Boreal coniferous forest (taiga)	VIII	14	23.0	0
Tundra	IX	5	8.2	1

Walter, H. *Vegetationszonen und Klima*. Eugen Ulmer: Stuttgart, Germany, **1970**

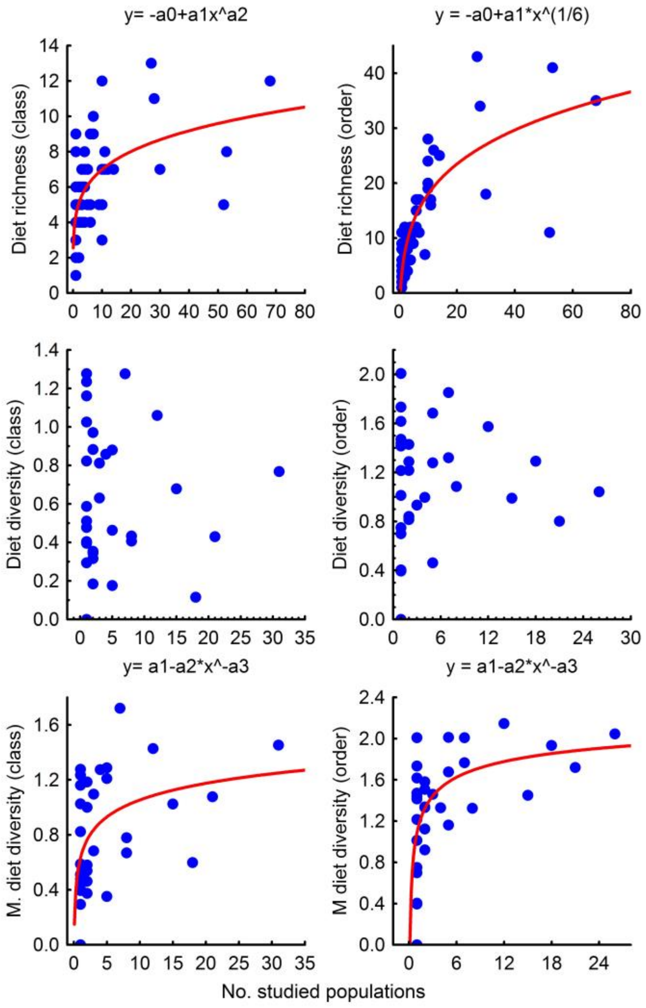

Figure S1. Accumulation curves of diet richness (top), diet diversity (middle) and maximum diet diversity (bottom) variables with respect to the number of studied populations at class (left) and order (right) taxonomic resolutions. Each point represents the mean diversity value of all the studied populations for each species.

Bibliographical list of sources used to determine the trophic niche of the Falconidae species.

Anderson, P. C., Kok, O. B., & Erasmus, B. H. (1999). Diet, body mass and condition of Lesser Kestrels Falco naumanni in South Africa. *Ostrich, 70*(2), 112.

Baker-Gabb, D. J. (1982). *Comparative ecology and behaviour of Swamp Harriers (Circus approximans), Spotted Harriers (Circus assimilis) and other raptors in Australia and New Zealand.* (Ph.D. dissertation), Monash University, Melbourne, Australia.

Baker-Gabb, D. J. (1983). Observations on the mating system and breeding success of the Marsh Harrier in coastal southeastern Australia. *Corella, 7*, 109-113.

Baker-Gabb, D. J. (1984). The feeding ecology and behaviour of seven species of raptor overwintering in coastal Victoria. *Australian Wildlife Research, 11*(3), 517-532.

Baker, A. J. (1998). *Status and breeding biology, ecology, and behavior of the Orange-breasted Falcon (Falco deiroleucus) in Guatemala and Belize.* (M.Sc. thesis), Brigham Young University, Provo, UT.

Baumgartner, A. M., & Baumgartner, F. M. (1944). Hawks and owls in Oklahoma 1939-1942: Food habits and population changes. *The Wilson Bulletin, 56*(4), 209-215.

Baziz, B., Souttou, K., Doumandji, S., & Denys, C. (2001). Quelques aspects sur le régime alimentaire du Faucon crécerelle *Falco tinnunculus* (Aves, Falconidae) en Algérie. *Alauda, 69*(3), 413-418.

Becker, D. M. (1985). Food habits of Richardson's Merlins in southeastern Montana. *The Wilson Bulletin, 97*(2), 226-230.

Biondi, L. M., B¢, M. S., & Favero, M. (2005). Diet of the Chimango Caracara (*Milvago chimango*) during the breeding season in the southeastern Buenos Aires Province, Argentina. *Ornitologia Neotropical, 16*(1), 31-42.

Bó, M. S. (1999). Dieta del halcón plomizo (*Falco femoralis*) en el sudeste de la Provincia de Buenos Aires, Argentina. *Ornitologia Neotropical, 10*, 95-99.

Booms, T. L., & Fuller, M. R. (2003). Gyrfalcon diet in central west Greenland during the nesting period. *The Condor, 105*(3), 528-537.

Boratyński, Z., & Kasprzyk, K. (2005). Does urban structure explain shifts in the food niche of the Eurasian Kestrel (*Falco tinnunculus*). *Buteo, 14*, 11-17.

Boyce, D. (1985). Prairie falcon prey in the Mojave Desert, California. *Journal of Raptor Research, 19*(4), 128-134.

Byre, V. J. (1990). A group of young Peregrine Falcons prey on migrating bats. *The Wilson Bulletin, 102*(4), 728-730.

Cade, T. J. (1960). *Ecology of the Peregrine and Gyrfalcon populations in Alaska*: University of California Press Berkeley, CA.

Cade, T. J., & Digby, R. D. (1982). *The falcons of the world*. New York: Harper Collins.

Carillo, J. (1994). Geographic variation in the spring diet of *Falco tinnunculus* L. on the islands of Fuerteventura and El Hierro (Canary Islands). *Bonner Zoologische Beiträge, 45*(1), 39-48.

Carter, K. M. (2003). *Food habits, reproductive status, habitat use, and behavior of Peregrine Falcon in Kentucky.* University of Kentucky.

Carvalho, C. E. A., Zorzin, G., Canuto, M., & Carvalho Filho, E. P. M. (2007). A preliminary study of the diet of the Bat Falcon (*Falco rufigularis*) in southwestern Brazil. In K. L. Bildstein, D. R. Barber, & A. Zimmerman (Eds.), *Neotropical raptors* (Vol. 1, pp. 275). Orwigsburg, PA: Hawk Mountain Sanctuary. (Reprinted from: IN FILE).

Castellanos, A., Argüelles, C., Salinas, F., RodrÍguez, A., Ortega-Rubio, A., & Morrison, J. L. (2006). Diet of breeding peregrine falcons at a coastal lagoon, Baja California Sur, Mexico. *Journal of Raptor Research, 40*(3), 241-244.

Castro Cabral, J., Granzinolli, M. M., & Mottajunior, M. (2006). Dieta do quiriquiri, *Falco sparverius* (Aves: Falconiformes), na Estação Ecológica de Itirapina, SP. *Revista Brasileira De Ornitologia, 14*(4), 393-399.

Catry, P., Lecoq, M., & Strange, I. J. (2008). Population growth and density, diet and breeding success of Striated Caracaras *Phalcoboenus australis* on New Island, Falkland Islands. *Polar Biology, 31*(10), 1167-1174.

Cavé, A. J. (1968). The breeding of the Kestrel, *Falco tinnunculus* L., in the reclaimed area Oostelijk Flevoland. *Netherlands Journal of Zoology, 18*(3), 313-407.

Clark, W. S. (1999). *A field guide to the raptors of Europe, the Middle East, and North Africa*. Oxford Oxford University Press.

Costantini, D., Casagrande, S., Di Lieto, G., Fanfani, A., & Dell'Omo, G. (2005). Consistent differences in feeding habits between neighbouring breeding kestrels. *Behaviour, 142*(9-10), 9-10.

Cotter, R. C., Boag, D. A., & Shank, C. C. (1992). Raptor predation on Rock Ptarmigan (*Lagopus mutus*) in the central canadian artic. *Journal of Raptor Research, 26*(3), 146-151.

Cramp, S., & Perrins, C. M. (1994). *The birds of the Western Palearctic*. Oxford: Oxford University Press.

Charter, M., Izhaki, I., Bouskila, A., Leshem, Y., & Penteriani, V. (2007). The effect of different nest types on the breeding success of Eurasian kestrels (*Falco tinnunculus*) in a rural ecosystem. *Journal of Raptor Research, 41*(2), 143-149.

Chavez-Ramirez, F., & Enkerlin, E. (1993). Notes on the food habits of the bat falcon (*Falco rufigularis*) in Tamaulipas, Mexico. *Journal of Raptor Research, 25*, 142-143.

Dawson, R. D., Mossop, D. H., & Boukall, B. (2011). Prey use and selection in relation to reproduction by Peregrine Falcons breeding along the Yukon River, Canada. *Journal of Raptor Research, 45*(1), 27-37.

De León, L., Rodríguez, B., Martín, A., Nogales, M., Alonso, J., & Izquierdo, C. (2007). Status, Distribution, and Diet of Eleonora's Falcon (*Falco eleonorae*) in the Canary Islands. *Journal of Raptor Research, 41*(4), 331-336.

de Roland, L.-A. R., Rabearivony, J., Razafimanjato, G., Robenarimangason, H., & Thorstrom, R. (2005). Breeding biology and diet of Banded Kestrels *Falco zoniventris* on Masoala Peninsula, Madagascar. *Ostrich-Journal of African Ornithology, 76*(1-2), 32-36.

de Vries, T., Black, J., Solis, C., & Hernandez, C. (1983). *Natural history of the Carunculated Caracara (Phalcoboenus carunculatus) in the paramos of Antisana and Cotopaxi, Ecuador*. Quito, Ecuador: Ediciones de la Universidad Católica.

Debus, S. J. S., Hatfield, T. S., Olde, G. S., & Rose, A. B. (2005). Breeding behaviour and diet of a pair of black falcons *Falco subniger* in northern New South Wales. *Australian Field Ornithology, 22*(4), 165-181.

Diaz del Campo, F. (1974). Some comments on the feeding of the Peregrine Falcon. *Ardeola, 19*, 351-357.

Dickman, C. R., Daly, S. E. J., & Connell, G. W. (1991). Dietary relationships of the barn owl and austalian kestrel on island off the coast of Western-Australia. *Emu, 91*(2), 69-72.

Donadio, E., Bolgeri, M. J., & Wurstten, A. (2007). First quantitative data on the diet of the Mountain Caracara (*Phalcoboenus megalopterus*). *Journal of Raptor Research, 41*(4), 328-330.

Drewitt, E. J. A., & Dixon, N. (2008). Diet and prey selection of urban-dwelling peregrine falcons in southwest England. *British Birds, 101*(2), 58-67.

DuVal, E. H., Greene, H. W., & Manno, K. L. (2006). Laughing Falcon (*Herpetotheres cachinnans)* Predation on Coral Snakes (*Micrurus nigrocinctus*). *Biotropica, 38*(4), 566-568.

De Silva, R. S. (1997). Ecology and behavior of wintering *Falco peregrinus* (Falconiformes: Falconidae) in southeastern Brazil. *Ararajuba, 5*(2), 203-208.

Egret, C. (2004). Observation of the chimango caracara (*Milvago chimango*) feeding on common lesser toads *(Bufo fernandezae*). *Journal of Raptor Research, 38*(2), 190-191.

El-Bahrawy, A. F., Reda, L., Cagañ, L., Shoaib, M., & Kamel, M. (2007). The kestrel (*Falco tinnunculus rupicolaeformis*) as a natural enemy of some agricultural pests in Ismailia governorate *Acta pytotechnica et zootechnica, 2*, 35-39.

Ellis, D., Sabo, B., Fackler, J., & Millsap, B. (2002). Prey of the Peregrine Falcon (*Falco peregrinus cassini*) in southern Argentina and Chile. *Journal of Raptor Research, 36*(4), 315-319.

Ellis, D. H., Ellis, C. H., Sabo, B. A., Rea, A. M., Dawson, J., Fackler, J. K., . . . Smith, D. G. (2004). Summer diet of the Peregrine Falcon in faunistically rich and poor zones of Arizona analyzed with capture-recapture modeling. *The Condor, 106*(4), 873-886.

Engh, A., Franklin, W., & Sarno, R. (1997). Breeding biology and food habits of the Andean Crested Caracara (*Polyborus plancus plancus*) in the Patagonia of southern Chile. *Vida Silvestre Neotrop, 6*, 48-58.

Ezaki, Y., & Mizota, H. (2006). Wintering of a Peregrine Falcon on an electricity pylon and its food in a suburban area of western Japan. *Ornithological Science, 5*(2), 211-216.

Fairley, I., & Mclean, A. (1965). Notes on the summer food of the Kestrel in Northern Ireland. *British Birds, 58*, 145-148.

Feare, C., Temple, S., & Procter, J. (1974). The status, distribution and diet of the Seychelles Kestrel *Falco araea*. *Ibis, 116*(4), 548-551.

Ferguson-Lees, J., & Christie, D. A. (2001). *Raptors of the world*. Singapore: Houghton Mifflin Harcourt.

Figueroa, R., & Corales, E. (2002). Winter diet of the American Kestrel (*Falco sparverius*) in the forested Chilean Patagonia, and its relation to the availability of prey. *International Hawkwatcher, 5*, 7-14.

Figueroa, R., & Corales, E. (2005). Seasonal diet of the Aplomado Falcon (*Falco femoralis*) in an agricultural area of Araucanía, southern Chile. *Journal of Raptor Research, 39*(1), 55-60.

Figueroa Rojas, R., Alvarado Orellana, S., & Corales Stappung, E. S. (2004). Notes on a range expansion and summer diet of the mountain caracara in the Andes of South-Central Chile. *Journal of Raptor Research, 38*(3), 290-292.

Figueroa Rojas, R. A., & Corales Stappung, E. S. (2004). Summer diet comparison between the American Kestrel (*Falco sparverius*) and Aplomado Falcon (*Falco femoralis*) in an agricultural area of Araucanía, southern Chile. *El hornero, 19*(2), 53-60.

Folgado, G. L. (2010). Dieta del Cernícalo vulgar (*Falco tinnunculus*) durante el periodo reproductor en una colonia suburbana al Este de la Península Ibérica. *El Serenet, 8*, 33-39.

Forsman, D. (1999). *The raptors of Europe and the Middle East: a handbook of field identification*. London: T & AD Poyser.

Forsman, D. (2007). *The Raptors of Europe and the Middle East: A Handbook of Field Identification*. London: Christopher Helm.

Franco, A., & Andrada, J. (1977). Alimentación y selección de presa en *Falco naumanni*. *Ardeola, 23*, 137-187.

Galetti, M., & Guimarães Jr, P. R. (2004). Seed dispersal of Attalea phalerata (Palmae) by Crested caracaras (*Caracara plancus*) in the Pantanal and a review of frugivory by raptors. *Ararajuba, 12*(2), 133-135.

Glutz v. Blotzheim, U., Bauer, K., & Bezzel, E. (1971). Handbuch der Vögel Mitteleuropas. Bd. 4.

Gomes, F. B. R., Crozariol, M. A., & Bispo, A. Â. (2008). Predação do cágado *Phrynops geoffroanus* (Chelonia: Pleurodira) pelo falcão *Milvago chimachim*a (Aves: Falconiformes), numa reserva florestal em Icem, SP. *Atualidades ornitológicas, 144*.

Grenci, S., & Di Vittorio, M. (2004). Alimentazione del Lanario *Falco biarmicus feldeggii* in Sicilia. *AVOCETTA, 28*(1), 93-95.

Groombridge, J. J., Nicoll, M., Jones, C. G., & Watson, J. (2004). *Associations of evolutionary and ecological distinctiveness amongst Indian Ocean kestrels.* Paper presented at the Proceedings of The World Working Group on Birds of Prey & Owls, Berlin.

Guhrs, P., & Osborne, T. (1988). *Prey selectivity of the Red-necked Falcon Falco chiquera in Luangwa Valley, Zambia.* Paper presented at the Proceedings of the Sixth Pan-African Ornithological Congress., Backhurst.

Hagen, Y. (1952). *Rovfuglene og viltpleien*: Gyldendal Norsk Forlag.

Hasler, K. (2004). *Biologie de la reproduction du faucon crécerelle (Falco tinnunculus) en milieu agricole.* Université de Neuchâtel.

Haverschmidt, F. (1962). Notes on the feeding habits and food of some hawks of Surinam. *The Condor, 64*, 154-158.

Hector, D. P. (1987). The decline of the aplomado falcon in the United States. *American Birds, 41*(3), 381-389.

Hector, P. (1985). The diet of the Aplomado Falcon (*Falco femoralis*) in eastern Mexico. *The Condor, 87*(3), 336-342.

Heddergott, M., Claussen, A., & Roth, E. (1998). Eurasian Hobby *Falco subbuteo* and Common Kestrel *Falco tinnunculus* as predator of bats (Chiroptera). *Abhandlungen und Berichte aus dem Museum Heineanum, 4*, 129-131.

Heintzelman, D. (2000). Yellow-headed caracara (*Milvago chimachima*) fishing in Amazonia. *International Hawkwatcher, 1*, 15.

Hoyo, J., Elliott, A., Sargatal, J., & Collar, N. J. (1994). *Handbook of the Birds of the World: New World vultures to Guinea fowl*. Barcelona: Lynx Edicions.

Hunt, L. E. (1994). *Diet and habitat use of nesting Prairie Falcons (Falco mexicanus) in an agricultural landscape in southern Alberta.* University of Alberta, Alberta.

Institute, W. H. M. (1999). *American kestrel (Falco sparverius)* (Vol. 3). Madison, MS: USDA, Natural Resources Conservation Service, Wildlife Habitat Management Institute.

Jenkins, A., & Avery, G. (1999). Diets of breeding peregrine and lanner falcons in South Africa. *Journal of Raptor Research, 33*(3), 190-206.

Jiménez, J. (1993). Notes on the diet of the aplomado falcon (*Falco femoralis*) in north central chile. *Journal of Raptor Research, 27*(3), 161-163.

Khaleghizadeh, A., & Sehhatisabet, M. E. (2006). Contribution to the knowledge of the diet of Iranian birds. *Áåðêóò*, 145.

Kok, O. B., Kok, A. C., & Van Ee, C. A. (2000). Diet of the migrant Lesser Kestrels *Falco naumanni* in their winter quarters in South Africa. *Acta Ornithologica, 35*(2), 147-151.

Kopij, G. (2002). Food of the Lesser Kestrel (*Falco naumanni*) in its winter quarters in South Africa. *Journal of Raptor Research, 36*(2), 148-152.

Kopij, G. (2005). The role of sun spiders (Solifugae) in the diet of wintering Lesser Kestrels (*Falco naumanni*). *Buteo, 14*, 19-22.

Kopij, G. (2007). Seasonal and annual dietary changes in Lesser Kestrels *Falco naumanni* wintering in Lesotho. *Ostrich-Journal of African Ornithology, 78*(3), 615-619.

Kopij, G. (2010). Seasonal variation in the diet of the Amur kestrel (*Falco amurensis*) in its winter quarter in Lesotho. *African Journal of Ecology, 48*(2), 559-562.

Korpimäki, E. (1985). Diet of the kestrel *Falco tinnunculus* in the breeding season. *Ornis Fennica, 62*(3), 130-137.

Korpimäki, E. (1986). Diet variation, hunting habitat and reproductive output of the kestrel *Falco tinnunculus* in the light of the optimal diet theory. *Ornis Fennica, 63*(3), 84-90.

Korpimäki, E. (1986). Seasonal-changes in the Food of the tengmalms ows *Aegolius funereus* in wester Finland. *Annales Zoologici Fennici, 23*(4), 339-344.

Kubler, S., Kupko, S., & Zeller, U. (2005). The kestrel (*Falco tinnunculus* L.) in Berlin: investigation of breeding biology and feeding ecology. *Journal of Ornithology, 146*(3), 271-278.

Leonardi, G., & Mannino, V. (2007). Feeding habits of urban Peregrine *Falco peregrinus brookei* in eastern Sicily. *AVOCETTA, 31*(1/2), 73.

Macías-Duarte, A., Montoya, A. B., Hunt, W. G., Lafón-Terrazas, A., Tafanelli, R., & Marti, C. (2004). Reproduction, prey, and habitat of the Aplomado Falcon (*Falco femoralis*) in desert grasslands of Chihuahua, Mexico. *The Auk, 121*(4), 1081-1093.

Marti, C. D., & Braun, C. E. (1975). Use of tundra habitats by Prairie Falcons in Colorado. *The Condor, 77*(2), 213-214.

Martín, M., Guerrero, M., mendoza, P., & Antolín, J. (2007). Experiencia con cámara web para la determinación del régimen alimenticio en la zepa "Iglesia de la Purificación" de Almendralejo extremadura. . *Primilla info, 6*, 11-13.

Martínez-Padilla, J. (2006). *Enciclopedia Virtual de los Vertebrados Espanoles Cernícalo vulgar–Falco tinnunculus Linnaeus, 1758*

Mayol, J. (1977). Estudios sobre el halcón de Eleonor, *Falco eleonorae,* en las islas Baleares. *Ardeola, 23*, 103-136.

Mayol, J. (1996). El Halcón de Eleonora (*Falco eleonorae*): situación de la especie y de su conocimiento. En, J. Muntaner & J. Mayol (Eds): Biología y Conservación de las Rapaces Mediterráneas, 1994. *Monografía*(4), 117-125.

McDonald, P. G., Baker-Gabb, D., & Warkentin, I. (2006). The breeding diet of different Brown Falcon (*Falco berigora*) pairs occupying the same territory over twenty years apart. *Journal of Raptor Research, 40*(3), 228-231.

McDonald, P. G., Olsen, P. D., & Baker-Gabb, D. J. (2003). Territory fidelity, reproductive success and prey choice in the brown falcon, *Falco berigora*: a flexible bet-hedger? *Australian Journal of Zoology, 51*(4), 399-414.

McNutt, J. W. (1981). *Selección de presa y comportamiento de caza del halcón peregrino (Falco peregrinus) en Magallanes y Tierra del Fuego.* Paper presented at the Anales del Instituto de la Patagonia.

Mearns, R. (1983). The diet of the Peregrine *Falco peregrinus* in south Scotland during the breeding season. *Bird Study, 30*(2), 81-90.

Mella, J. (2002). Dieta del cernícalo (*Falco sparverius*) y del Tucúquere (*Bubo magellanicus*) en un ambiente cordillerano de Chile central. *Boletín Chileno de Ornitología, 9*(1), 34-37.

Monteiro-Filho, E. (1995). Fishing behavior of yellow-headed caracara, *Milvago chimachima*(Falconidae) in southeast Brazil. *Ciencia e cultura(Sao Paulo), 47*(1), 86-87.

Montoya, A. B., Zwank, P. J., & Cardenas, M. (1997). Breeding Biology of Aplomado Falcons in Desert Grasslands of Chihuahua, Mexico (Biología Reproductiva de *Falco femoralis* en los Yerbasales Desérticos de Chihuahua, México). *Journal of Field Ornithology, 68*(1), 135-143.

Morata, G., & de Donan, E. B. (1971). Observaciones sobre la reproducción del alcotán (*Falco subbuteo*). *Ardeola, 15*, 37-48.

Morimando, F., & Pezzo, F. (1997). Lanner falcon (*Falco biarmicus feideggi*) in central Italy. *The Journal of raptor research, 31*(1), 40-43.

Morrison, J. L., Pias, K. E., Abrams, J., Gottlieb, I. G., Deyrup, M., & McMillian, M. (2008). Invertebrate diet of breeding and nonbreeding Crested Caracaras (*Caracara cheriway)* in Florida. *Journal of Raptor Research, 42*(1), 38-47.

Movalli, P. (2000). Heavy metal and other residues in feathers of laggar falcon *Falco biarmicus jugger* from six districts of Pakistan. *Environmental Pollution, 109*(2), 267-275.

Muir, D., & Bird, D. M. (1984). Food of gyrfalcons at a nest on Ellesmere Island. *The Wilson Bulletin, 96*(3), 464-467.

Negro, J. J., Ibáñez, C., Pérezjordá, J. L., & Delariva, M. (1992). Winter predation by common kestrel *Falco tinnunculus* on Pipistrelle bats *Pipistrellus pipistrellus* in southern Spain. *Bird Study, 39*(3), 195-199.

Nelson, K., & Mo, C. L. (1996). Olive Ridley (*Lepidochelys olivacea*) nests excavated by caracaras (*Polyborus plancus*) at Nancite Beach. *Marine Turtle Newsletter, 74*, 10-11.

Newton, I., Meek, E., & Little, B. (1984). Breeding season foods of merlins *Falco columbarius* in Northumbria. *Bird Study, 31*(1), 49-56.

Nicholls, M., & Clarke, R. (1991). *Biology and Conservation of Small Falcons*. University of Kent press: University of Kent.

Nielsen, O. K. (1999). Gyrfalcon predation on ptarmigan: numerical and functional responses. *Journal of Animal Ecology, 68*(5), 1034-1050.

Notarnicola, J., & Seipke, S. H. (2004). Prey of Peregrine Falcons *Falco peregrinus* in the city of La Plata, Argentina *Primer Simposio Argentino sobre Investigaci¢n y Conservaci¢n de Rapaces - SAICR I, Museo de La Plata, 1 y 2 Octubre 2004, Libro de Acta [Proceedings of the First Argentine Symposium on Investigation and Conservation of Raptors - SAICR I]* (pp. 15-16). Buenos Aires, Argentina: Museo de la Plata. (Reprinted from: IN FILE).

Nunnery, T., & Welford, M. R. (2002). Barred Forest-Falcon (*Micrastur ruficollis)* predation on a hummingbird. *Journal of Raptor Research, 36*(3), 239-240.

Nystrom, J. (2004). *Predator-prey interactions of raptors in an arctic community.* University of Stockholm, Stockholm.

Nyström, J., Ekenstedt, J., Engström, J., & Angerbjörn, A. (2005). Gyr Falcons, ptarmigan and microtine rodents in northern Sweden. *Ibis, 147*(3), 587-597.

Ogden, V. T., & Hornocker, M. G. (1977). Nesting density and success of prairie falcons in southwestern Idaho. *The Journal of Wildlife Management, 41*(1), 1-11.

Olmos, F., Pacheco, J. F., & Silveira, L. F. (2006). Notes on Brazilian birds of prey. *Revista Brasileira De Ornitologia, 14*(4), 401-404.

Olmos, F., & Sazima, I. (2009). Fishing behaviour by Black Caracaras (*Daptrius ater*) in the Amazon. *Biota Neotropica, 9*(3), 399-401.

Olsen, J., Fuentes, E., Bird, D. M., Rose, A., & Judge, D. (2008). Dietary shifts based upon prey availability in Peregrine Falcons and Australian Hobbies breeding near Canberra, Australia. *Journal of Raptor Research, 42*(2), 125-137.

Ontiveros, D. (2005). Abundance and diet of Alexander's Kestrel (*Falco tinnunculus alexandri*) on Boavista Island (Archipelago of Cape Verde). *Journal of Raptor Research, 39*(1), 80-83.

Oro, D., & Tella, J. L. (1995). A comparison of two methods for studying the diet of the peregrine falcon. *Journal of Raptor Research, 29*(3), 207-210.

Palmer, A. G., Nordmeyer, D. L., & Roby, D. D. (2004). Nestling provisioning rates of Peregrine Falcons in interior Alaska. *Journal of Raptor Research, 38*(1), 9-18.

Parker, M. N. (1997). *Ecology of nesting laughing falcons and bat falcons in Tikal National Park, Guatemala: foraging and niche breadth.* Boise State University, Boise.

Parr, S. (1985). The breeding ecology and diet of the Hobby Falco subbuteo in southern England. *Ibis, 127*(1), 60-73.

Parrott, D., Henderson, I., Deppe, C., & Whitfield, P. (2008). Scottish racing pigeons killed by Peregrine Falcons *Falco peregrinus*: estimation of numbers from ring recoveries and Peregrine daily food intake: Capsule Daily food intake gave the best indication, with 7–23% of the pigeon population estimated to have been taken. *Bird Study, 55*(1), 32-42.

Perez-Granados, C. (2010). Diet of adult lesser kestrels *Falco naumanni* during the breding season in central Spain. *Ardeola, 57*(2), 443-448.

Pierson, J., & Donahue, P. (1983). Peregrine Falcon feeding on bats in Suriname, South America. *American Birds, 37*(3), 257-259.

Platt, J. B. (1976). Gyrfalcon nest site selection and winter activity in the western Canadian arctic. *Canadian Field Naturalist, 90*(3), 338-345.

Poole, K. G., & Boag, D. (1988). Ecology of gyrfalcons, *Falco rusticolus*, in the central Canadian Arctic: diet and feeding behaviour. *Canadian Journal of Zoology, 66*(2), 334-344.

Purger, J. (1998). Diet of Red-footed Falcon *Falco vespertinus* nestlings from hatching to fledging. *Ornis Fennica, 75*, 185-191.

Quinn, J. L. (1997). The effects of hunting Peregrines *Falco peregrinus* on the foraging behaviour and efficiency of the Oystercatcher *Haematopus ostralegus*. *Ibis, 139*(1), 170-173.

Rand, A. L. (1936). The distribution and habits of Madagascar birds. *Bulletin of the American Museum of Natural History, 72*, 143-500.

Rand, A. L., & Gilliard, E. T. (1967). *Handbook of New Guinea birds*. London: Weidenfeld and Nicolson.

Ratcliffe, D. (1993). *The peregrine falcon*. London: A&C Black.

Razafimanjato, G., de Roland, L.-A. R., Rabearivony, J., & Thorstrom, R. (2007). Nesting biology and food habits of the Peregrine Falcon *Falco peregrinus* radama in the south-west and central plateau of Madagascar. *Ostrich-Journal of African Ornithology, 78*(1), 7-12.

Richmond, A. R. (1976). Feeding of nestlings by the Caracara in Costa Rica. *Wilson Bulletin, 88*(4), 667.

Riegert, J., Loevy, M., & Fainova, D. (2009). Diet composition of Common Kestrels *Falco tinnunculus* and Long-eared Owls *Asio otus* coexisting in an urban environment. *Ornis Fennica, 86*(4), 123-130.

Rizzolli, F., Sergio, F., Marchesi, L., & Pedrini, P. (2005). Density, productivity, diet and population status of the Peregrine Falcon *Falco peregrinus* in the Italian Alps: Capsule The population of Peregrine Falcon in the central-eastern Italian Alps is much larger than previously thought. *Bird Study, 52*(2), 188-192.

Robbins, M. B., & D.A., W. (1982). Observations at a laughing falcon *Herpetotheres cachinnans* nest. *Wilson Bull, 94*(1), 83-84.

Rocha, P. A. (1998). Dieta e comportamento alimentar do Peneireiro-de-dorso-liso *Falco naumanni*. *Airo, 9*(1/2), 40-47.

Rodríguez-Estrella, R., & Rivera-Rodríguez, L. B. (1997). Crested Caracara food habits in the Cape region of Baja California, Mexico. *Journal of Raptor Research, 31*(3), 228-233.

Rodríguez, C. (2004). *Factores ambientales relacionados con el exito reproductivo del Cernicalo Primilla. Cambio climatico e intensificacion agraria.* University of Salamanca, Salamanca.

Rodríguez, C., Tapia, L., Kieny, F., & Bustamante, J. (2010). Temporal changes in lesser kestrel (*Falco naumanni*) diet during the breeding season in southern Spain. *Journal of Raptor Research, 44*(2), 120-128.

Roma, S., & Rossetti, M. (1998). Notes about the predation of hobby *Falco subbuteo* on Chiroptera sp. [Ulteriori osservazioni sul lodolaio, Falco subbuteo, che preda chirotteri.]. *Uccelli d'Italia, 23*(1-2), 38-38.

Rosenfield, R. N., Schneider, J. W., Papp, J. M., & Seegar, W. S. (1995). Prey of Peregrine Falcons breeding in west Greenland. *The Condor, 97*(3), 763-770.

Santillán, M., Travaini, A., Zapata, S. C., RodrÍguez, A., Donázar, J., Procopio, D. E., & Zanón, J. I. (2009). Diet of the American Kestrel in Argentine Patagonia. *Journal of Raptor Research, 43*(4), 377-381.

Santillan, M. Á., Travaini, A., & Fernández, J. (2010). Dieta del Halcón peregrino (*Falco peregrinus*) en la ría Deseadp, Patagonia Austal Argentina. *Boletín Chileno de Ornitología, 16*(1), 1-8.

Sazima, I. (2007). The jack-of-all-trades raptor: versatile foraging and wide trophic role of the Southern Caracara (*Caracara plancus*) in Brazil, with comments on feeding habits of the Caracarini. *Revista Brasileira De Ornitologia, 15*(4), 592-597.

Sazima, I., & Olmos, F. (2009). The Chimango Caracara (*Milvago chimango*), an additional fisher among Caracarini falcons. *Biota Neotropica, 9*(3), 403-405.

Seaton, R., Hyde, N., Holland, J. D., Minot, E. O., & Springett, B. P. (2008). Breeding season diet and prey selection of the New Zealand falcon (*Falco novaeseelandiae*) in a plantation forest. *Journal of Raptor Research, 42*(4), 256-264.

Seijas, A. E. (1996). Feeding of the bat falcon *(Falco rufigularis*) in an urban environment. *Journal of Raptor Research, 30*(1), 33-35.

Sergio, F., & Bogliani, G. (1999). Eurasian hobby density, nest area occupancy, diet, and productivity in relation to intensive agriculture. *The Condor, 101*(4), 806-817.

Serra, G., Lucentini, M., & Romano, S. (2001). Diet and prey selection of nonbreeding Peregrine Falcons in an urban habitat of Italy. *Journal of Raptor Research, 35*(1), 61-64.

Sherrod, S. K. (1978). Diet of North American Falconiformes. *Journal of Raptor Research, 12*(3/4), 49.

Sielicki, J., Mizera, T., & Group, E. P. F. W. (2009). *Peregrine Falcon populations: status and perspectives in the 21st century*: Turul Pub.

Silvano, R., & Rossetti, M. (1999). Notes about the predation of hobby *Falco subbuteo* on Chiroptera sp Ulteriori osservazioni sul lodolaio, *Falco subbuteo*, che preda chirotteri. *Uccelli d'Italia, 231*(2), 38.

Simonetti, J., Nuñez, H., & Yañez, J. (1982). *Falco sparverius* L: rapaz generalista en Chile central (Aves: Falconidae). *Boletín del Museo Nacional de Historia Natural, 39*, 119-124.

Sodhi, N. S., & Oliphant, L. W. (1993). Prey selection by urban-breeding Merlins. *The Auk, 110*(4), 727-735.

Souttou, K., Baziz, B., Doumandji, S., Denys, C., & Brahimi, R. (2007). Prey selection in the common kestrel, *Falco tinnunculus* (Aves, Falconidae) in the Algiers suburbs (Algeria). *Folia Zoologica -Praha, 56*(4).

Spina, F., & Leonardi, G. (2007). Italian action plan for Eleonora's falcon (*Falco eleonorae*). [Piano d'azione nazionale per il falco della regina (Falco eleonorae).]. *Quaderni di Conservazione della Natura, 26*, 1-69.

Squires, J. R., Anderson, S., & Oakleaf, R. (1989). Food habits of nesting prairie falcons in campbell county in Campbell Country, Wyoming. *Journal of Raptor Research, 23*(4), 157-161.

Stephenson, A. (2001). *Ecology and breeding biology of Lanner Falcons in the Eastern Cape Province, South Africa.* Rhodes University, Grahamstown

Tatum, J. (1981). Peregrine fishing at sea. *British Birds, 74*(2), 97-97.

Tejero, E., Soler, M., Camacho, I., & Avila, J. M. (1982). Contribucion al conocimiento del regimen alimenticio del cernicalo primilla (*Falco naumanni*, Fleisch., 1758). *Boletin de la Estacion Central de Ecologia, 11*, 77-82.

Thiollay, J. M. (1998). The diurnal raptors (Falconiformes) of the Reserve Speciale d'Anjanaharibe-Sud: abundance, distribution, and conservation. In S. M. Goodman (Ed.), *A floral and faunal inventory of the Reserve Speciale d'Anjanaharibe-Sud, Madagascar: with reference to elevational variation* (Vol. 90, pp. 129-138). (Reprinted from: IN FILE).

Thompson, G., & Beaumont, D. (2003). Peregrine eating moths. *British Birds, 96*(3), 138-138.

Thorstrom, R. (1996). Fruit-eating behavior of a Barred Forest-falcon. *Journal of Raptor Research, 30*(1), 44-44.

Thorstrom, R., Ramos, J. D., & Castillo, J. M. (2000). Breeding biology and behavior of the Collared Forest-Falcon (*Micrastur semitorquatus*) in Guatemala. *Ornitologia Neotropical, 11*, 1-12.

Travaini, A., Donázar, J. A., Ceballos, O., & Hiraldo, F. (2001). Food habits of the Crested Caracara (*Caracara plancus*) in the Andean Patagonia: the role of breeding constraints. *Journal of arid environments, 48*(2), 211-219.

Travaini, A., Donázar, J. A., Rodríguez, A., Ceballos, O., Funes, M., Delibes, M., & Hiraldo, F. (1998). Use of European hare (Lepus europaeus) carcasses by an avian scavenging assemblage in Patagonia. *Journal of Zoology, 246*(2), 175-181.

Valdez, U. (1996). *Characterization and comparison of the diets of the Laughing Falcon (Herpetotheres cachinnans) and Crested Caracara (Polyborus plancus) in Coto de Caza El Angolo and surroundings.* (Undergraduate thesis), Universidad Nacional Agraria La Molina, Lima, Peru.

Valverde, J. A. (1967). *Estructura de una comunidad mediterránea de vertebrados terrestres*. Madrid: Consejo Superior de Investigaciones Científicas.

Van Zyl, A. (1994). A comparison of the diet of the Common kestrel *Falco tinnunculus* in South Africa and Europe. *Bird Study, 41*(2), 127-130.

Vargas, R. J., Bó, M. S., Favero, M., & Morrison, J. L. (2007). Diet of the southern caracara (*Caracara plancus*) in Mar Chiquita Reserve, southern Argentina. *Journal of Raptor Research, 41*(2), 113-121.

Village, A. (1982). The diet of kestrel in relation to vole abundance. *Bird Study, 29*(2), 129-138.

Village, A. (1990). *The kestrel* (T. A. D. Poyser Ed.). London: T. & A.D. Poyser.

Walter, H. S. (1997). Ecology of American Kestrels Wintering on Socorro Island Mexico. *Journal of Raptor Research, 31*(4), 384-384.

Watson, J. (1979). Food of Merlins nesting in young conifer forest. *Bird Study, 26*(4), 253-258.

Watson, J. (1981). *Population ecology, food and conservation of the Seychelles kestrel (Falco araea) on Mahé.* University of Aberdeen.

Wauer, R. H. (2005). *The American Kestrel: Falcon of Many Names*. Boulder: Big Earth Publishing.

Whitacre, D., Ukrain, D., & Falxa, G. (1983). Notes on the hunting behavior and diet of the crested caracara in northeastern Chiapas and Tabasco, Mexico. *The Wilson Bulletin, 94*(4), 565-566.

White, C. M., & Roseneau, D. G. (1970). Observations on food, nesting, and winter populations of large North American falcons. *The Condor, 72*, 113-115.

White, C. M., & Springer, H. K. (1965). Notes on the Gyrfalcon in western coastal Alaska. *The Auk, 82*(1), 104-105.

Wink, M., Biebach, H., Feldmann, F., Scharlau, W., Swatschek, I., Wink, C., & Ristow, D. (1993). Contribution to the breeding biology of Eleonora's Falcon (*Falco eleonorae*). In M. K. Nicholls & R. Clarke (Eds.), *Biology and conservation of small falcons* (pp. 59-72). London: Hawk and Owl Trust. (Reprinted from: IN FILE).

Winkel, E. (2007). The endemic kestrels of the Cape Verde Islands. *Bulletin of the African Bird Club, 14*(1), 81-83.

Yanez, J. L. (1980). Diet and weight of American Kestrel in central Chile. *The Auk, 97*(3), 629-631.

Yanez, J. L., Nunez, H., & Jaksic, F. M. (1982). Food habits and weight of Chimango Caracaras in Central Chile. *The Auk, 99*(1), 170-171.

Yosef, R., & Yosef, D. (1992). Hunting behavior of audubons Crested caracara. *Journal of Raptor Research, 26*(2), 100-101.

Zilio, F. (2006). Diet of *Falco sparverius* Linnaeus 1758 (Aves: Falconidae) and *Athene cunicularia* (Molina, 1782) (Aves: Strigidae) in a dune region on north coast of Rio Grande do Sul, Brazil. *Revista Brasileira De Ornitologia, 14*(4), 379-392.

Zmihorski, M., Żmihorski, M., & Rejt, Ł. (2007). Weather-Dependent Variation in the Cold-Season Diet of Urban Kestrels *Falco tinnunculus*. *Acta Ornithologica, 42*(1), 107-113.

R-Scripts

```
falco.tree <- read.tree("Falconidae_Fuchs.phy")


data <- read.table("datos falconidae correg2.txt", header=TRUE, row.names=1 )

dataNA <- read.table("datos falconidae correg22.txt", header=TRUE, row.names=1 )

data <- read.table("datos falconidae correg2residualsfilogenia.txt",
header=TRUE,row.names=1 )

dataNA<-
mydata(data$Sp,data$BSI,data$N_studies,data$Richness_Order,data$Richness_Class)

model.lambda<-pgls(indep1 ~ 1, data = data , lambda='ML')

hd<-lm(RangoShannonO~NPobO ,data=data)

summary(hd)

myresiduals<-residuals(hd)

write.table(myresiduals, row.names=TRUE,"rango residuals ORDEN.txt")

hd<-lm(RangoShannonC~NPobC ,data=data)

summary(hd)

myresiduals<-residuals(hd)

resid<-myresiduals

write.table(myresiduals, row.names=TRUE,"rango residuals famili.txt")

hd<-lm(XShannonC~NPobC ,data=data)

summary(hd)

myresiduals<-residuals(hd)

write.table(myresiduals, row.names=TRUE,"X residuals famili.txt")

hd<-lm(XshannonO~NPobO ,data=data)

summary(hd)

myresiduals<-residuals(hd)

write.table(myresiduals, row.names=TRUE,"X residuals orden.txt")
```

```
hd<-lm(MaxShannonC~NPobC ,data=data)
summary(hd)
myresiduals<-residuals(hd)
write.table(myresiduals, "MAx residuals famili.txt")


hd<-lm(MaxShannonO~NPobO ,data=data)
summary(hd)
myresiduals<-residuals(hd)
write.table(myresiduals, "MAx residuals orden.txt")
hd<-lm(MinShannonC~NPobC ,data=data)
summary(hd)
myresiduals<-residuals(hd)
write.table(myresiduals, "Min residuals famili.txt")
hd<-lm(MinShannonO~NPobO ,data=data)
summary(hd)
myresiduals<-residuals(hd)
write.table(myresiduals, "Min residuals orden.txt")
hd<-lm(Richness_Class~N_studies ,data=data)
summary(hd)
myresiduals<-residuals(hd)
write.table(myresiduals, "Richness residuals famili.txt")
hd<-lm(Richness_Order~N_studies ,data=data)
summary(hd)
myresiduals<-residuals(hd)
write.table(myresiduals, "Richness residuals orden.txt")
mod3 <- pgls(RangoShannonO~NPobO ,data=data,lambda='ML')
```

```
##### lambdas ####

RIQRO <- (data$RIQRO)

RIQRF<- (data$RIQRF)

residXO <- (data$residXO)

residXC <- (data$residXC)

residRO <- (data$residRO)

residRF <- (data$residRF)

residMAO <- (data$residMAO)

residMAC <- (data$residMAC)

residMIO <- (data$residMIO)

residMIC <- (data$residMIC)

XShannonC <- (data$XShannonC)

XshannonO <- (data$XshannonO)

BSI <- (data$BSI)

km_total <- (data$km_total)

km_total2 <- (log(data$km_total))

names(data)

names(km_total2)<-names(km_total)<-names(BSI)<-names(XShannonC)<-
names(XshannonO)<-names(residMIC) <-names(residMIO) <- names(residMAC) <-
names(residMAO) <- names(residRF) <-names(residRO) <- names(residXC)<-names(residXO) <-
names(RIQRF) <- names(RIQRO) <- rownames(data)


phylosig(falco.tree, residMIO, method="lambda",test=TRUE)

phylosig(falco.tree, residMIC, method="lambda",test=TRUE)

phylosig(falco.tree, residMAO, method="lambda",test=TRUE)

phylosig(falco.tree, residMAC, method="lambda",test=TRUE)

phylosig(falco.tree,residRO , method="lambda",test=TRUE)

phylosig(falco.tree, residRF, method="lambda",test=TRUE)
```

```
phylosig(falco.tree,residXO , method="lambda",test=TRUE)

phylosig(falco.tree, residXC, method="lambda",test=TRUE)

phylosig(falco.tree,RIQRO , method="lambda",test=TRUE)

phylosig(falco.tree, RIQRF, method="lambda",test=TRUE)

phylosig(falco.tree,XshannonO , method="lambda",test=TRUE)

phylosig(falco.tree, XShannonC, method="lambda",test=TRUE)

phylosig(falco.tree, residMIO, method="lambda")

phylosig(falco.tree, residMIC, method="lambda")

phylosig(falco.tree, residMAO, method="lambda")

phylosig(falco.tree, residMAC, method="lambda")

phylosig(falco.tree,residRO , method="lambda")

phylosig(falco.tree, residRF, method="lambda")

phylosig(falco.tree,residXO , method="lambda")

phylosig(falco.tree, residXC, method="lambda")

phylosig(falco.tree,RIQRO , method="lambda")

phylosig(falco.tree, RIQRF, method="lambda")

phylosig(falco.tree,XshannonO , method="lambda")

phylosig(falco.tree, XShannonC, method="lambda")

phylosig(falco.tree,BSI , method="lambda")

phylosig(falco.tree, km_total, method="lambda")

phylosig(falco.tree, km_total2, method="lambda")



#### phylogeny number of populations studied####


Richness_Class <- (data$Richness_Class)

Richness_Order <- (data$Richness_Order)
```

```
MaxShannonO <- (data$MaxShannonO)

MaxShannonC <- (data$MaxShannonC)

XShannonC <- (data$XShannonC)

XshannonO <- (data$XshannonO)

N_studies <- (data$N_studies)

NPobC <- (data$NPobC)

NPobO <- (data$NPobO)


names(data)

names(NPobC)<-names(NPobO)<-names(N_studies)<-names(MinShannonO)<-
names(MinShannonC)<-names(MaxShannonC)<-names(MaxShannonO)<-
names(XshannonO)<-names(Richness_Order) <-names(XShannonC)<- names(Richness_Class)
<- rownames(data)


p=AllTrees[1]

B=readORDER(p)

#Actually compute the ED (ie ES) score

ESS=(ES_v2(B[[1]],B[[2]],B[[3]]))^(-1)

# sort it properly

ess=(ESS[falco.tree$tip.label,])

DR <- ess

DR <- DR[falco.tree$tip.label]

NPobC <- NPobC[falco.tree$tip.label]

NPobO <- NPobO[falco.tree$tip.label]

N_studies <- N_studies[falco.tree$tip.label]

XShannonC <- XShannonC[falco.tree$tip.label]

XshannonO <- XshannonO[falco.tree$tip.label]

MaxShannonC <- MaxShannonC[falco.tree$tip.label]

MaxShannonO <- MaxShannonO[falco.tree$tip.label]
```

```
Richness_Order <- Richness_Order[falco.tree$tip.label]

Richness_Class <- Richness_Class[falco.tree$tip.label]

DF.falcon <-
data.frame(taxa=falco.tree$tip.label,DR,NPobC,NPobO,N_studies,XShannonC,XshannonO,MinShannonO,MinShannonC,MaxShannonC,MaxShannonO,Richness_Order,Richness_Class)

formula <- Richness_Class~N_studies

formula2 <-Richness_Order~N_studies

formula3 <-XshannonO~NPobO

formula5 <-MaxShannonO~NPobO

formula31 <-XShannonC~NPobC

formula51<-MaxShannonC~NPobC

cdat <- comparative.data(data=DF.falcon,phy=falco.tree,names.col=taxa,vcv=T, na.omit=F, scope=formula)

mod1 <- pgls(formula,cdat,lambda='ML')

mod2 <- pgls(formula2,cdat,lambda='ML')

mod3 <- pgls(formula3,cdat,lambda='ML')

mod4 <- pgls(formula4,cdat,lambda='ML')

mod5 <- pgls(formula5,cdat,lambda='ML')

mod31 <- pgls(formula31,cdat,lambda='ML')

mod41<- pgls(formula41,cdat,lambda='ML')

mod51 <- pgls(formula51,cdat,lambda='ML')

#Rrichness

summary(mod1)

summary(mod2)

#Orden: mean, max, min

summary(mod3)

summary(mod4)

summary(mod5)

#class; mean max min
```

```
summary(mod31)

summary(mod41)

summary(mod51)




##### #################    phylogenetic analyses  ################

##############################   *****     BSI  ******* ############################


#### BSI maximo      ####

## order ##

indep1 <- (data$BSI)

indep2<- (data$residMAO)

indep3 <- log(data$km_total)

names(data)

names(indep3) <-names(indep1) <- names(indep2) <- rownames(data)

p=AllTrees[1]

B=readORDER(p)

#Actually compute the ED (ie ES) score

ESS=(ES_v2(B[[1]],B[[2]],B[[3]]))^(-1)

# sort it properly

ess=(ESS[falco.tree$tip.label,])

DR <- ess

DR <- DR[falco.tree$tip.label]

indep1 <- indep1[falco.tree$tip.label]## esto ordena las variables igual para crear el
data.frame

indep2 <- indep2[falco.tree$tip.label]

indep3 <- indep3[falco.tree$tip.label]
```

```
DF.falcon <- data.frame(taxa=falco.tree$tip.label,DR,indep1,indep2,indep3,indep4,indep5)

formula <- indep1~indep2+indep3

cdat <- comparative.data(data=DF.falcon,phy=falco.tree,names.col=taxa,vcv=T, na.omit=F, scope=formula)

mod1 <- pgls(formula,cdat,lambda='ML')


summary(mod1)

myresiduals<-residuals(mod1)

shapiro.test(myresiduals)

lillie.test(myresiduals)



##  Class ##

indep1 <- (data$BSI)

indep2<- (data$residMAC)

indep3 <- log(data$km_total)



names(data)

names(indep3) <-names(indep1) <- names(indep2) <- rownames(data)

p=AllTrees[1]

B=readORDER(p)

#Actually compute the ED (ie ES) score

ESS=(ES_v2(B[[1]],B[[2]],B[[3]]))^(-1)

# sort it properly

ess=(ESS[falco.tree$tip.label,])

DR <- ess

DR <- DR[falco.tree$tip.label]
```

```
indep1 <- indep1[falco.tree$tip.label]## esto ordena las variables igual para crear el data.frame

indep2 <- indep2[falco.tree$tip.label]

indep3 <- indep3[falco.tree$tip.label]

DF.falcon <- data.frame(taxa=falco.tree$tip.label,DR,indep1,indep2,indep3)

formula <- indep1~indep2+indep3

cdat <- comparative.data(data=DF.falcon,phy=falco.tree,names.col=taxa,vcv=T, na.omit=F, scope=formula)

mod1 <- pgls(formula,cdat,lambda='ML')


summary(mod1)

myresiduals<-residuals(mod1)

shapiro.test(myresiduals)

lillie.test(myresiduals)




#### BSI	mean diversity####


## order##


indep1 <- (data$BSI)

indep2<- (data$XshannonO)

indep3 <- log(data$km_total)
```

```
names(data)

names(indep3) <-names(indep1) <- names(indep2) <- rownames(data)



p=AllTrees[1]

B=readORDER(p)

#Actually compute the ED (ie ES) score

ESS=(ES_v2(B[[1]],B[[2]],B[[3]]))^(-1)

# sort it properly

ess=(ESS[falco.tree$tip.label,])

DR <- ess

DR <- DR[falco.tree$tip.label]

indep1 <- indep1[falco.tree$tip.label]## esto ordena las variables igual para crear el
data.frame

indep2 <- indep2[falco.tree$tip.label]

indep3 <- indep3[falco.tree$tip.label]



DF.falcon <- data.frame(taxa=falco.tree$tip.label,DR,indep1,indep2,indep3)

formula <- indep1~indep2+indep3

cdat <- comparative.data(data=DF.falcon,phy=falco.tree,names.col=taxa,vcv=T, na.omit=F,
scope=formula)

mod1 <- pgls(formula,cdat,lambda='ML')



summary(mod1)

myresiduals<-residuals(mod1)

shapiro.test(myresiduals)
```

```
lillie.test(myresiduals)

mod1


### class##


indep1 <- (data$BSI)

indep2<- (data$XShannonC)

indep3 <- log(data$km_total)



names(data)

names(indep3) <-names(indep1) <- names(indep2) <- rownames(data)



p=AllTrees[1]

B=readORDER(p)

#Actually compute the ED (ie ES) score

ESS=(ES_v2(B[[1]],B[[2]],B[[3]]))^(-1)

# sort it properly

ess=(ESS[falco.tree$tip.label,])

DR <- ess

DR <- DR[falco.tree$tip.label]

indep1 <- indep1[falco.tree$tip.label]## esto ordena las variables igual para crear el
data.frame

indep2 <- indep2[falco.tree$tip.label]

indep3 <- indep3[falco.tree$tip.label]

DF.falcon <- data.frame(taxa=falco.tree$tip.label,DR,indep1,indep2,indep3)

formula <- indep1~indep2+indep3
```

```
cdat <- comparative.data(data=DF.falcon,phy=falco.tree,names.col=taxa,vcv=T, na.omit=F,
scope=formula)

mod1 <- pgls(formula,cdat,lambda='ML')



summary(mod1)

myresiduals<-residuals(mod1)

shapiro.test(myresiduals)

lillie.test(myresiduals)

mod1



####    BSI richness   ####

## order ##

indep1 <- (data$BSI)

indep2<- (data$RIQRO)

indep3 <- log(data$km_total)



names(data)

names(indep3) <-names(indep1) <- names(indep2) <- rownames(data)



p=AllTrees[1]

B=readORDER(p)

#Actually compute the ED (ie ES) score

ESS=(ES_v2(B[[1]],B[[2]],B[[3]]))^(-1)

# sort it properly
```

```
ess=(ESS[falco.tree$tip.label,])

DR <- ess

DR <- DR[falco.tree$tip.label]

indep1 <- indep1[falco.tree$tip.label]## esto ordena las variables igual para crear el data.frame

indep2 <- indep2[falco.tree$tip.label]

indep3 <- indep3[falco.tree$tip.label]


DF.falcon <- data.frame(taxa=falco.tree$tip.label,DR,indep1,indep2,indep3)

formula <- indep1~indep2+indep3

cdat <- comparative.data(data=DF.falcon,phy=falco.tree,names.col=taxa,vcv=T, na.omit=F, scope=formula)

mod1 <- pgls(formula,cdat,lambda='ML')


summary(mod1)

myresiduals<-residuals(mod1)

shapiro.test(myresiduals)

lillie.test(myresiduals)




##  Class ##

indep1 <- (data$BSI)

indep2<- (data$RIQRF)

indep3 <- log(data$km_total)

names(data)

names(indep3) <-names(indep1) <- names(indep2) <- rownames(data)

p=AllTrees[1]
```

```
B=readORDER(p)

#Actually compute the ED (ie ES) score

ESS=(ES_v2(B[[1]],B[[2]],B[[3]]))^(-1)

# sort it properly

ess=(ESS[falco.tree$tip.label,])

DR <- ess

DR <- DR[falco.tree$tip.label]

indep1 <- indep1[falco.tree$tip.label]## esto ordena las variables igual para crear el data.frame

indep2 <- indep2[falco.tree$tip.label]

indep3 <- indep3[falco.tree$tip.label]




DF.falcon <- data.frame(taxa=falco.tree$tip.label,DR,indep1,indep2,indep3)

formula <- indep1~indep2+indep3

cdat <- comparative.data(data=DF.falcon,phy=falco.tree,names.col=taxa,vcv=T, na.omit=F, scope=formula)

mod1 <- pgls(formula,cdat,lambda='ML')




phylosig(falco.tree, indep2, method="lambda",test=TRUE)

summary(mod1)

myresiduals<-residuals(mod1)

shapiro.test(myresiduals)

lillie.test(myresiduals)
```

```
################### ***range size***
################################################

#### range size mean diversity ####

## order##

indep1 <- (data$BSI)

indep2<- (data$XshannonO)

indep3 <- (log(data$km_total))

names(data)

names(indep3) <-names(indep1) <- names(indep2) <- rownames(data)

p=AllTrees[1]

B=readORDER(p)

#Actually compute the ED (ie ES) score

ESS=(ES_v2(B[[1]],B[[2]],B[[3]]))^(-1)

# sort it properly

ess=(ESS[falco.tree$tip.label,])

DR <- ess

DR <- DR[falco.tree$tip.label]

indep1 <- indep1[falco.tree$tip.label]## esto ordena las variables igual para crear el
data.frame

indep2 <- indep2[falco.tree$tip.label]

indep3 <- indep3[falco.tree$tip.label]

DF.falcon <- data.frame(taxa=falco.tree$tip.label,DR,indep1,indep2,indep3,indep4,indep5)

formula2 <-indep3~indep2

cdat <- comparative.data(data=DF.falcon,phy=falco.tree,names.col=taxa,vcv=T, na.omit=F,
scope=formula)
```

```
mod2 <- pgls(formula2,cdat,lambda='ML')

summary(mod2)

par(mfrow=c(2,2))

plot(mod1)

dev.off()

myresiduals<-residuals(mod2)

shapiro.test(myresiduals)

lillie.test(myresiduals)




###class##

indep1 <- (data$BSI)

indep2<- (data$XShannonC)

indep3 <- (log(data$km_total))

names(data)

names(indep3) <-names(indep1) <- names(indep2) <- rownames(data)

p=AllTrees[1]

B=readORDER(p)

#Actually compute the ED (ie ES) score

ESS=(ES_v2(B[[1]],B[[2]],B[[3]]))^(-1)

# sort it properly

ess=(ESS[falco.tree$tip.label,])

DR <- ess

DR <- DR[falco.tree$tip.label]

indep1 <- indep1[falco.tree$tip.label]## esto ordena las variables igual para crear el
data.frame

indep2 <- indep2[falco.tree$tip.label]
```

```
indep3 <- indep3[falco.tree$tip.label]

DF.falcon <- data.frame(taxa=falco.tree$tip.label,DR,indep1,indep2,indep3,indep4,indep5)

formula2 <-indep3~indep2

cdat <- comparative.data(data=DF.falcon,phy=falco.tree,names.col=taxa,vcv=T, na.omit=F,
scope=formula)

mod2 <- pgls(formula2,cdat,lambda='ML')

summary(mod2)

myresiduals<-residuals(mod2)

shapiro.test(myresiduals)

lillie.test(myresiduals)





##################### **** BSI resi filo ***** ########


#### Resid phylo max diversity  BSI     ####

## order ##

indep1 <- (data$BSI)

indep2<- (data$filoresMAO)

indep3 <- log(data$km_total)

names(data)

names(indep3) <-names(indep1) <- names(indep2) <- rownames(data)

p=AllTrees[1]

B=readORDER(p)

#Actually compute the ED (ie ES) score

ESS=(ES_v2(B[[1]],B[[2]],B[[3]]))^(-1)
```

```
# sort it properly

ess=(ESS[falco.tree$tip.label,])

DR <- ess

DR <- DR[falco.tree$tip.label]

indep1 <- indep1[falco.tree$tip.label]## esto ordena las variables igual para crear el data.frame

indep2 <- indep2[falco.tree$tip.label]

indep3 <- indep3[falco.tree$tip.label]


DF.falcon <- data.frame(taxa=falco.tree$tip.label,DR,indep1,indep2,indep3,indep4,indep5)

formula <- indep1~indep2+indep3

cdat <- comparative.data(data=DF.falcon,phy=falco.tree,names.col=taxa,vcv=T, na.omit=F, scope=formula)

mod1 <- pgls(formula,cdat,lambda='ML')


summary(mod1)

myresiduals<-residuals(mod1)

shapiro.test(myresiduals)

lillie.test(myresiduals)



## Class ##

indep1 <- (data$BSI)

indep2<- (data$filoresMAF)

indep3 <- log(data$km_total)

names(data)

names(indep3) <-names(indep1) <- names(indep2) <- rownames(data)

p=AllTrees[1]
```

```
B=readORDER(p)

#Actually compute the ED (ie ES) score

ESS=(ES_v2(B[[1]],B[[2]],B[[3]]))^(-1)

# sort it properly

ess=(ESS[falco.tree$tip.label,])

DR <- ess

DR <- DR[falco.tree$tip.label]

indep1 <- indep1[falco.tree$tip.label]## esto ordena las variables igual para crear el data.frame

indep2 <- indep2[falco.tree$tip.label]

indep3 <- indep3[falco.tree$tip.label]

DF.falcon <- data.frame(taxa=falco.tree$tip.label,DR,indep1,indep2,indep3)

formula <- indep1~indep2+indep3

cdat <- comparative.data(data=DF.falcon,phy=falco.tree,names.col=taxa,vcv=T, na.omit=F, scope=formula)

mod1 <- pgls(formula,cdat,lambda='ML')



summary(mod1)

myresiduals<-residuals(mod1)

shapiro.test(myresiduals)

lillie.test(myresiduals)



#### phylo resi bsi RICHNESS ####

## order ##

indep1 <- (data$BSI)
```

```
indep2<- (data$filoresRIQO)

indep3 <- log(data$km_total)

names(data)

names(indep3) <-names(indep1) <- names(indep2) <- rownames(data)

p=AllTrees[1]

B=readORDER(p)

#Actually compute the ED (ie ES) score

ESS=(ES_v2(B[[1]],B[[2]],B[[3]]))^(-1)

# sort it properly

ess=(ESS[falco.tree$tip.label,])

DR <- ess

DR <- DR[falco.tree$tip.label]

indep1 <- indep1[falco.tree$tip.label]## esto ordena las variables igual para crear el
data.frame

indep2 <- indep2[falco.tree$tip.label]

indep3 <- indep3[falco.tree$tip.label]

DF.falcon <- data.frame(taxa=falco.tree$tip.label,DR,indep1,indep2,indep3)

formula <- indep1~indep2+indep3

cdat <- comparative.data(data=DF.falcon,phy=falco.tree,names.col=taxa,vcv=T, na.omit=F,
scope=formula)

mod1 <- pgls(formula,cdat,lambda='ML')


summary(mod1)

myresiduals<-residuals(mod1)

shapiro.test(myresiduals)

lillie.test(myresiduals)

mod1
```

```
## Class ##

indep1 <- (data$BSI)

indep2<- (data$filoresiRIF)

indep3 <- log(data$km_total)

names(data)

names(indep3) <-names(indep1) <- names(indep2) <- rownames(data)

p=AllTrees[1]

B=readORDER(p)

#Actually compute the ED (ie ES) score

ESS=(ES_v2(B[[1]],B[[2]],B[[3]]))^(-1)

# sort it properly

ess=(ESS[falco.tree$tip.label,])

DR <- ess

DR <- DR[falco.tree$tip.label]

indep1 <- indep1[falco.tree$tip.label]## esto ordena las variables igual para crear el
data.frame

indep2 <- indep2[falco.tree$tip.label]

indep3 <- indep3[falco.tree$tip.label]

DF.falcon <- data.frame(taxa=falco.tree$tip.label,DR,indep1,indep2,indep3)

formula <- indep1~indep2+indep3

cdat <- comparative.data(data=DF.falcon,phy=falco.tree,names.col=taxa,vcv=T, na.omit=F,
scope=formula)

mod1 <- pgls(formula,cdat,lambda='ML')


phylosig(falco.tree, indep2, method="lambda",test=TRUE)

summary(mod1)

myresiduals<-residuals(mod1)
```

```
shapiro.test(myresiduals)

lillie.test(myresiduals)

mod1


########################   **** range size resid phylogeny ***** #######


####  phylo resid range size max diversity ####

##  order ##

indep1 <- (data$BSI)

indep2<- (data$filoresMAO)

indep3 <- (log(data$km_total))


names(data)

names(indep3) <-names(indep1) <- names(indep2) <- rownames(data)

p=AllTrees[1]

B=readORDER(p)

#Actually compute the ED (ie ES) score

ESS=(ES_v2(B[[1]],B[[2]],B[[3]]))^(-1)

# sort it properly

ess=(ESS[falco.tree$tip.label,])

DR <- ess

DR <- DR[falco.tree$tip.label]

indep1 <- indep1[falco.tree$tip.label]## esto ordena las variables igual para crear el
data.frame

indep2 <- indep2[falco.tree$tip.label]

indep3 <- indep3[falco.tree$tip.label]


DF.falcon <- data.frame(taxa=falco.tree$tip.label,DR,indep1,indep2,indep3)
```

```
formula2 <-indep3~indep2

cdat <- comparative.data(data=DF.falcon,phy=falco.tree,names.col=taxa,vcv=T, na.omit=F, scope=formula)

mod2 <- pgls(formula2,cdat,lambda='ML')


summary(mod2)

myresiduals<-residuals(mod2)

shapiro.test(myresiduals)

lillie.test(myresiduals)


## Class ##

indep1 <- (data$BSI)

indep2<- (data$filoresMAF)

indep3 <- (log(data$km_total))

names(data)

names(indep3) <-names(indep1) <- names(indep2) <- rownames(data)

p=AllTrees[1]

B=readORDER(p)

#Actually compute the ED (ie ES) score

ESS=(ES_v2(B[[1]],B[[2]],B[[3]]))^(-1)

# sort it properly

ess=(ESS[falco.tree$tip.label,])

DR <- ess

DR <- DR[falco.tree$tip.label]

indep1 <- indep1[falco.tree$tip.label]## esto ordena las variables igual para crear el data.frame

indep2 <- indep2[falco.tree$tip.label]

indep3 <- indep3[falco.tree$tip.label]
```

```
DF.falcon <- data.frame(taxa=falco.tree$tip.label,DR,indep1,indep2,indep3)

formula2 <-indep3~indep2

cdat <- comparative.data(data=DF.falcon,phy=falco.tree,names.col=taxa,vcv=T, na.omit=F,
scope=formula)

mod2 <- pgls(formula2,cdat,lambda='ML')


summary(mod2)

myresiduals<-residuals(mod2)

shapiro.test(myresiduals)

lillie.test(myresiduals)






##### phylo Resid Richness Area ########

##########    Order ###

indep1 <- (data$BSI)

indep2<- (data$filoresRIQO)

indep3 <- (log(data$km_total))

names(data)

names(indep3) <-names(indep1) <- names(indep2) <- rownames(data)

p=AllTrees[1]

B=readORDER(p)

#Actually compute the ED (ie ES) score

ESS=(ES_v2(B[[1]],B[[2]],B[[3]]))^(-1)

# sort it properly

ess=(ESS[falco.tree$tip.label,])
```

```
DR <- ess

DR <- DR[falco.tree$tip.label]

indep1 <- indep1[falco.tree$tip.label]## esto ordena las variables igual para crear el data.frame

indep2 <- indep2[falco.tree$tip.label]

indep3 <- indep3[falco.tree$tip.label]

DF.falcon <- data.frame(taxa=falco.tree$tip.label,DR,indep1,indep2,indep3)

formula2 <-indep3~indep2

cdat <- comparative.data(data=DF.falcon,phy=falco.tree,names.col=taxa,vcv=T, na.omit=F, scope=formula)

mod2 <- pgls(formula2,cdat,lambda='ML')

summary(mod2)

myresiduals<-residuals(mod2)

shapiro.test(myresiduals)

lillie.test(myresiduals)




## CLASS    ##

indep1 <- (data$BSI)

indep2<- (data$RIQRF)

indep3 <- (log(data$km_total))

names(data)

names(indep3) <-names(indep1) <- names(indep2) <- rownames(data)

p=AllTrees[1]

B=readORDER(p)

#Actually compute the ED (ie ES) score

ESS=(ES_v2(B[[1]],B[[2]],B[[3]]))^(-1)
```

```
# sort it properly

ess=(ESS[falco.tree$tip.label,])

DR <- ess

DR <- DR[falco.tree$tip.label]

indep1 <- indep1[falco.tree$tip.label]## esto ordena las variables igual para crear el data.frame

indep2 <- indep2[falco.tree$tip.label]

indep3 <- indep3[falco.tree$tip.label]

DF.falcon <- data.frame(taxa=falco.tree$tip.label,DR,indep1,indep2,indep3)

formula2 <-indep3~indep2

cdat <- comparative.data(data=DF.falcon,phy=falco.tree,names.col=taxa,vcv=T, na.omit=F, scope=formula)

mod2 <- pgls(formula2,cdat,lambda='ML')

summary(mod2)

myresiduals<-residuals(mod2)

shapiro.test(myresiduals)

lillie.test(myresiduals)


#######################   **** range size + BSI ***** #######


indep1 <- (data$BSI)

indep3 <- (log(data$km_total))


names(data)

names(indep3) <-names(indep1)  <- rownames(data)

p=AllTrees[1]

B=readORDER(p)

#Actually compute the ED (ie ES) score
```

```
ESS=(ES_v2(B[[1]],B[[2]],B[[3]]))^(-1)

# sort it properly

ess=(ESS[falco.tree$tip.label,])

DR <- ess

DR <- DR[falco.tree$tip.label]

indep1 <- indep1[falco.tree$tip.label]## esto ordena las variables igual para crear el data.frame

indep3 <- indep3[falco.tree$tip.label]


DF.falcon <- data.frame(taxa=falco.tree$tip.label,DR,indep1,indep2,indep3)

formula2 <-indep1~indep3

cdat <- comparative.data(data=DF.falcon,phy=falco.tree,names.col=taxa,vcv=T, na.omit=F, scope=formula)

mod2 <- pgls(formula2,cdat,lambda='ML')


summary(mod2)

myresiduals<-residuals(mod2)

shapiro.test(myresiduals)

lillie.test(myresiduals)
```
